# Amodiaquine and Ciprofloxacin Combination in Plasmodiasis Therapy

**DOI:** 10.1155/2015/947390

**Published:** 2015-09-29

**Authors:** Peace Mayen Edwin Ubulom, Chinweizu Ejikeme Udobi, Mark Iheukwumere Madu

**Affiliations:** Pharmaceutical Microbiology and Parasitology Unit, Department of Pharmaceutics and Pharmaceutical Technology, Faculty of Pharmacy, University of Uyo, Uyo, Akwa Ibom, Nigeria

## Abstract

*Objective*. The study was designed to determine the efficacy of combined Amodiaquine and Ciprofloxacin in plasmodiasis therapy. *Method*. The in vivo antiplasmodial effect of different dosage levels of Amodiaquine, Ciprofloxacin, and their combinations against *Plasmodium berghei berghei* was evaluated using Swiss albino mice. *Results*. Amodiaquine (a known antiplasmodial agent) had a fairly significant antiplasmodial effect reducing the parasites for every 100 red blood cells (RBC) from 66 to 16 (75.75%) at the tolerable dosage level of 7.5 mg/kg body weight while Ciprofloxacin (an antibiotic known to have antimalarial effect) showed an insignificant antiplasmodial effect reducing the parasites for every 100 RBC from 65 to 64 (1.53%) at the tolerable dosage level of 10.7 mg/kg body weight. Conversely, the combination therapy of Amodiaquine and Ciprofloxacin had a significant antiplasmodial effect at all the doses administered. The combination of 7.5 mg/kg of Amodiaquine and 12.8 mg/kg of Ciprofloxacin, however, showed the most significant antiplasmodial effect of the doses used reducing the number of parasites per 100 RBC from 60 to 10 (83.33%). *Conclusions*. Appropriate Amodiaquine and Ciprofloxacin combination will be effective for the treatment of malaria and better than either Amodiaquine or Ciprofloxacin singly at their recommended dosage levels.

## 1. Introduction

Malaria remains one of the major killer diseases of the world. It causes illness by the ability of the causative parasite to invade the red blood cells [1] and the liver where they multiply. In extreme infections, up to 80% of the red blood cells can be parasitized and destroyed [2]. This massive cell destruction is known to lead to severe anaemia and clogging of the blood circulation of vital organs particularly the brain and eventually death.

Antimalarials are agents used to inhibit the development of* Plasmodium* (the causative parasite of malaria) and they are administered so they can completely destroy these parasites. There are quite a wide range of antimalarials available. They may be classified according to the different stages of the parasites they affect or according to their chemical nature and function [3].

All efforts made so far to eradicate malaria using these agents and very highly effective residual insecticides against Mosquito have failed [4]. This is basically due to the increasing resistance of Mosquito to insecticides and the parasites to the drugs. One of the major challenges of the medical world remains the eradication of malaria due to many reasons among which is resistance to antiplasmodial agents.

The need to achieve a more radical cure of the malaria scourge remains with us considering the continuous development of resistance to known and existing antimalarials being reported in different parts of the world. Currently, the World Health Organization guidelines for the treatment of malaria include the combination of one antimalarial and one antibiotic provided that there is evidence of their efficacy and safety [5]. Combined therapy which will help in tackling this challenge has therefore been recommended and efforts are on to find appropriate combinations to be used.

Combination therapy with antimalarial drug is the simultaneous use of two or more schizonticidal drugs with independent mode of action and different biochemical targets in the parasites. These can either be fixed where the drugs to be combined are coformulated in the same tablet or capsule or non-fixed, where they are coadministered in separate tablets or capsules. Drug combinations are used to exploit the synergistic and additive potentials of each drug as well as helping to improve efficacy while retarding the development of resistance to the individual components [6].

Amodiaquine is a semisynthetic aminoquinoline closely related to chloroquine. It acts by binding to the parasite and preventing the production of DNA and RNA and, subsequently, the synthesis of protein. Amodiaquine remains useful in the treatment of malaria in some parts of the world even though it has been reported to be linked to serum transferase elevation in about 1% of patients.

Ciprofloxacin is a second generation broad spectrum antibacterial agent. It has been reported to have good potentials for use as an antimalarial [7] and is known to target both the liver and blood parasite stages [8]. Though Ciprofloxacin may be well absorbed and may efficiently enter erythrocytes, its use as an antimalarial alone has been discouraged. This is because Ciprofloxacin antiplasmodial effect occurs only at an unacceptable high dose where the required serum concentration can be achieved [9] and a prolonged treatment regimen will have to be employed [7].

This paper presents, the results of efforts to determine in vivo the antiplasmodial effect of Amodiaquine and Ciprofloxacin combination therapy on* Plasmodium berghei berghei*.

## 2. Materials and Methods

*Plasmodium berghei berghei* strain obtained from the National Institute for Medical Research (NIMR) Lagos, Nigeria, was used for this study. It was maintained at the Animal House of the Department of Pharmacology and Toxicology, Faculty of Pharmacy, University of Uyo, Nigeria, by serial passage of blood from one mouse to the other intraperitoneally.

### 2.1. Drugs

Amodiaquine and Ciprofloxacin tablets used were obtained from a retail pharmacy outlet in Uyo, Nigeria.

### 2.2. Experimental Animals

Swiss albino mice (15–31 g) of either sex were used. They were adequately fed and kept in clean cages at the animal house of the Department of Pharmacology and Toxicology, Faculty of Pharmacy, University of Uyo, Nigeria. Approval for the use of the animals was given by the Animal Ethics Committee of the Faculty of Pharmacy, University of Uyo, Nigeria.

### 2.3. Inoculation of Experimental Animals

Each of the experimental animals was first confirmed to be free from any malarial parasites before inoculation. 0.2 mL of blood infected with* Plasmodium berghei berghei* was then used to inoculate each mice. Parasitaemia levels in each mice were determined after three days by taking blood samples from their tails and preparing Leishman's stained thin smears. The number of parasites was counted per 100 red blood cells.

### 2.4. Preparation of Drugs for Administration

#### 2.4.1. Amodiaquine

A known quantity of Amodiaquine tablet was powdered using a laboratory mortar and pestle. A stock solution of 30 mg/mL was made by dissolving 300 mg of the powder in 10 mLs of water. From this, further concentrations which would be tolerated by the experimental animals were made.

#### 2.4.2. Ciprofloxacin

One tablet of Ciprofloxacin (500 mg) was powdered using a laboratory mortar and pestle. The powder obtained was dissolved in 10 mLs of distilled water to obtain a concentration of 50 mg/mL. Further concentrations which would be tolerated by the experimental animals were further made from this.

### 2.5. Experimental Animal Treatment

Four days after infection of the experimental animals with* Plasmodium berghei berghei*, they were divided into four groups of 20 mice per group. Each group was designated to be treated with a specific drug or drug combination (e.g., group A was treated with Amodiaquine alone and group B was treated with Ciprofloxacin alone while group C was treated with combinations of Amodiaquine and ciprofloxacin). These first three groups A, B, and C were each further divided into five subgroups (A_1_–A_5_, B_1_–B_5,_ and C_1_–C_5_) with five mice in each group and they were treated with different doses of the drug or the drug combinations designated for the group. The last group, D, was not subdivided and served as the control group. See Table 4.

## 3. Results

Three days after the prescreened mice were infected with* Plasmodium berghei berghei*, signs of plasmodiasis were observed. There was increase in body temperature and decline in feeding rate. There was also marked reduction in movement and other activities generally.

Signs of recovery from the burden of parasitaemia were however observed after treatment with different concentrations of our drug combinations. These signs which included fall in body temperature, improvement in feeding, movement, and other activities of the animals were observed when the level of parasitaemia had reduced to about 30 parasites per 100 RBC after treatment.

The group of animals, which, though infected with the parasite, were not treated with the drugs, served as the control. They showed no signs of recovery from the burden of parasitaemia. Eventually, before the end of the laboratory work, all the animals died (Table 4).

Table 1 shows the parasitaemia levels and percentages of the reduction from day 1 to 3 after the administration of the drug combinations while Tables 2 and 3 show daily decreases in mean parasitaemia levels in the infected mice after treatment with only Amodiaquine (Table 2) and only Ciprofloxacin (Table 3).

The control (Table 4) shows that there was a reasonable increase in the mean parasitaemia level daily with the highest on the third day.

## 4. Discussion

It has been suggested that combination therapy holds the key to the future of malaria treatment. This is probably the reason why the search for effective antimalarials that can be used in combinations is being intensified. Drug combinations accelerate therapeutic response, protect the component drugs against resistance, and shorten duration of use thereby improving compliance [10].

Results obtained in this work showed that Ciprofloxacin when combined in appropriate proportions with Amodiaquine can be used in the control of Plasmodiasis. When Amodiaquine and Ciprofloxacin were used differently, they were found to be deficient in the adequate control of the parasite (Tables 2 and 3).

A combination of 7.5 mg/kg of Amodiaquine and 12.8 mg/kg of Ciprofloxacin caused an 83.3% reduction in the number of parasites from 60 to 10 within the period of study (Table 1). The percentage reduction achieved every day in the study using the combinations, when compared with the individual agents, showed a remarkable difference confirming an improvement in efficacy while the control (Table 4) which showed a steady increase in mean parasitaemia level confirmed that the reduction in parasitaemia level was due to the drugs.

Scientists before now have reported the positive effect of some antimalarial drug combinations with Ciprofloxacin [6, 10, 11]. It may, therefore, be suggested that Ciprofloxacin which has a low activity against* Plasmodium* may be enhancing the activity of the antimalarial.

The exact mechanism by which these agents work in combination was not studied. This remains an area of interest to us in our continuous studies of antibacterial and antiplasmodial combinations and interactions.

Results obtained goes to confirm along with results obtained elsewhere that though Ciprofloxacin may on its own not be a good antimalarial agent; it may be enhancing the activity of other antimalarials through a mechanism not yet known. It is also seen that combining Amodiaquine with Ciprofloxacin in the treatment of plasmodiasis is better than the use of Amodiaquine alone (in places where Amodiaquine is still dependent upon the treatment of malaria due to* Plasmodium*).

## Figures and Tables

**Table 1:** Results showing parasitaemia levels and percentage reduction of parasites three days after administration of different combinations of Amodiaquine and Ciprofloxacin in mice infected with *Plasmodium  berghei  berghei*.

Drug dosage mg/kg	Parasitaemia level (Per 100 RBC)	
	Before drug administration	Three days after drug administration
Amodiaquine (7.1 mg/kg) + Ciprofloxacin (10 mg/kg)	65	30 (53.84%)
Amodiaquine (7.2 mg/kg) + Ciprofloxacin (10.7 mg/kg)	65	23 (64.61%)
Amodiaquine (7.3 mg/kg) + Ciprofloxacin (11.4 mg/kg)	65	16 (75.38%)
Amodiaquine (7.4 mg/kg) + Ciprofloxacin (12.1 mg/kg)	65	13 (80.0%)
Amodiaquine (7.5 mg/kg) + Ciprofloxacin (12.8 mg/kg)	60	10 (83.3%)

Figures in brackets show percentage reduction after three days of drug administration.

**Table 2:** Results showing daily decreases in the mean parasitaemia level of mice treated with Amodiaquine along with their corresponding percentages.

Dose of Amodiaquine administered (Mg/mL)	Parasitaemia level per 100 RBC (percentage reduction)			
	Day 0	Day 1	Day 2	Day 3
7.1	66	62 (6.06%)	56 (15.15%)	38 (42.42%)
7.2	65	55 (15.38%)	43 (33.84%)	30 (53.84%)
7.3	67	57 **(**14.92%)	44 (34.32%)	28 (58.20%)
7.4	65	49 (24.61%)	35 (46.15%)	23 (64.61%)
7.5	66	49 (25.75%)	32 (51.51%)	19 (71.21%)

Figures in brackets are differences in parasitaemia level between the day (1, 2, or 3) and before treatment (day 0) expressed in percentage.

**Table 3:** Results showing daily decreases in the mean parasitaemia level of mice treated with Ciprofloxacin along with their corresponding percentages.

Dose of Ciprofloxacin administered (Mg/mL)	Parasitaemia level per 100 RBC (percentage reduction)			
	Day 0	Day 1	Day 2	Day 3
10.0	66	66 (0.00%)	66 (0.00%)	66 (0.00%)
10.7	65	65 (0.00%)	65 (0.00%)	64 (1.53%)
11.4	67	67 (0.00%)	67 (0.00%)	65 (2.98%)
12.1	65	63 (3.07%)	49 (24.61%)	41 (36.92%)
12.8	66	60 (9.09%)	46 (30.30%)	28 (57.57%)

Figures in brackets are differences in parasitaemia level between the day (1, 2, or 3) and before treatment (day 0) expressed in percentage.

**Table 4:** Results showing daily increases in the mean parasitaemia level of mice in the control group.

Dose (Mg/mL)	Parasitaemia level (per 100 RBC)			
	Day 0	Day 1	Day 2	Day 3
0	60	70	80	97
0	60	65	70	80
0	65	70	80	97
0	60	70	80	97
0	60	70	80	97
